# Reflectance-Only Retrieval of Refractive Index and Extinction Coefficient Dispersions in an Absorbing Thin Film via a Paul-Wavelet Repetition-Frequency Method

**DOI:** 10.1021/acsomega.6c00253

**Published:** 2026-09-04

**Authors:** Emre Coskun, Cansu Emir, Erhan Tiryaki, Makbule Terlemezoglu, Mehmet Parlak

**Affiliations:** † Department of Physics, 52984 Çanakkale Onsekiz Mart University, Çanakkale 17100, Turkey; ‡ Physics Group, Atilim University, Ankara 06830, Turkey; § Graduate School of Nature and Applied Science, Gazi University, Ankara 06500, Turkey; ∥ Marelli MAKO Turkey Elektrik Sanayi ve Ticaret A.Ş, Bursa 16220, Turkey; ⊥ Department of Physics, Gazi University, Ankara 06560, Turkey; # Department of Physics, Middle East Technical University, Ankara 06800, Turkey

## Abstract

Accurate knowledge of the refractive index (*n*) and extinction coefficient (*κ*) dispersions is essential for understanding the optical response of thin-film materials used in photovoltaics, optoelectronics, and related photonic applications. However, transmission-based dispersion extraction becomes unreliable for turbid, colloidal, nanostructured, or strongly absorbing thin films, where low transmission or scattering prevents accurate spectral evaluation. In this work, we introduce a reflectance-only framework based on the Paul wavelet transform applied to normal-incidence reflectance spectra. The method exploits repetition frequency analysis of interference fringes to retrieve continuous dispersions of *n* and *κ* without requiring a predefined dispersion model. The wavelet order provides explicit control over the joint spectral-Fourier resolution, allowing optimization for a given data set. The approach is validated through simulation studies and a noisy signal test with 10% additive random noise, and benchmarked against Minkov’s reflectance-based envelope/extrema method. Under noisy conditions, the proposed method preserves the refractive index trend close to the reference behavior, whereas the envelope-based approach shows larger deviations. The extinction coefficient is more sensitive to noise in both methods, although the wavelet-based retrieval remains comparatively stable. Experimental validation on a CdS thin film demonstrates consistency of the refractive index dispersion with literature data, while deviations in the extinction coefficient are attributed to its sensitivity to absorption-related and microstructural variations. The method requires independent film thickness information, as in all interference-based approaches, but is otherwise nondestructive and model-free, making it suitable for a broad range of thin-film systems including doped semiconductors, colloidal nanostructures, and hybrid organic–inorganic materials where conventional transmission or ellipsometric methods are difficult to apply.

## Graphical abstract

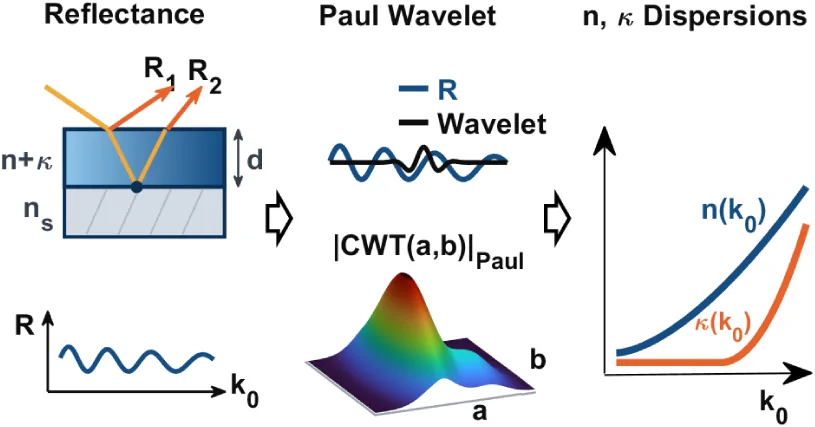

## 1 Introduction

Accurate and continuous determination of the refractive index (*n*) and extinction coefficient (*κ*) of absorbing thin films across the visible spectrum is crucial for the design and optimization of optoelectronic and photonic devices.^1–4^ Given this importance, a broad range of characterization strategies has been developed. Among them, transfer-matrix analysis of normal-incidence reflectance/transmittance spectra is a well-established route to retrieve the spectral dispersion of *n* and *κ*;^5^ however, the method is model-dependent and highly sensitive to the absolute magnitude and noise of the reflectance and transmittance spectra, which can lead to significant deviations in the retrieved optical constants.^6^ Swanepoel improved the spectral analysis by exploiting the envelopes formed by the interference fringes in the transmittance spectrum, enabling the determination of the values of both *n* and κ for weakly absorbing films on transparent substrates.^7^ While this noncontact optical approach is among the earliest and most recognized techniques based on fringe extrema, it requires well-resolved fringes and lateral uniformity.^7^ Today, spectroscopic ellipsometry has become one of the most widely used techniques for extracting the optical dispersion of thin films due to its nondestructive nature, high sensitivity, and ability to simultaneously determine film thickness and optical constants over a broad spectral range.^8^ Advanced implementations such as Mueller-matrix ellipsometry further extend its applicability to anisotropic and depolarizing samples. However, ellipsometric analysis generally relies on predefined dispersion models (e.g., Cauchy, Tauc–Lorentz, or Sellmeier), and the accuracy of the extracted optical constants may depend on the suitability of the selected model.^8^ In contrast, the frequency-domain wavelet framework proposed in this work retrieves the *n* and κ dispersions directly from the interference-fringe periodicity of the reflectance spectrum, without requiring a parametric dispersion model. Therefore, the proposed approach provides a complementary and computationally efficient alternative, particularly in cases where model selection is ambiguous or transmission-based measurements are unreliable. For the sub-bandgap, very-low-absorption regime, photothermal methods, most notably photothermal deflection spectroscopy (PDS), are employed to determine these optical constants owing to their superior sensitivity to weak absorption.^9^ The main drawbacks are the high cost of the instrumentation, the need for precise thermal and optical alignment, and the overall complexity of data acquisition and analysis.^9^

In our previous studies, a new approach was introduced in the literature. In these works, fringe repetition frequency in the transmittance spectrum was utilized to determine the spectral dispersion of *n* and *κ*.^10–13^ This frequency-based analysis provides significant advantages, including high noise immunity, a noncontact measurement configuration, short computation time, and ease of implementation. All of these methods, however, rely on the analysis of transmittance spectra. Unlike our previous Paul wavelet studies, which were confined to transmittance spectra and did not address κ retrieval, the present work extends the repetition-frequency framework to reflectance spectra and introduces simultaneous *n* and κ retrieval within this framework for the first time. In parallel, Minkov proposed empirical reflectance-based procedures in 1989 and 1991 to determine optical parameters directly from the reflectance spectrum of thin films.^14,15^ More broadly, reflectance-only retrieval strategies have been demonstrated across a wide range of spectral domains where transmission measurements are impractical or inaccessible. In the extreme ultraviolet (EUV), where materials are essentially opaque and transmission-based analysis is infeasible, optical constants have been determined solely from reflectance data using synchrotron-based reflectometry.^16,17^ A conceptually similar reflectance-only approach has proven effective for organic molecular monolayers (films so thin that their transmission signal is negligibly small) where spectroscopic reflectometry yields reliable dispersion of the optical functions and film thickness.^18,19^ The present study extends this broader family of reflectance-only characterization methods to the visible range by combining the Paul wavelet transform with repetition-frequency analysis.

In this study, we address scenarios where transmission measurements become unreliable, such as in turbid, colloidal, or highly absorbing films in which strong scattering or minimal transmission prevents accurate spectral evaluation, by introducing a reflectance-only characterization framework. We apply the Paul wavelet transform to the reflectance spectrum and use repetition-frequency analysis to determine the dispersion of *n* and *κ*. The Paul wavelet, used in the Continuous Wavelet Transform (CWT), affords tunable joint resolution in the spectral and Fourier domains, which makes it particularly well suited to capturing interference-fringe periodicities in reflectance data.^20^ The algorithm has been implemented and validated through simulation studies, including controlled noise injections to assess robustness and precision. Finally, we demonstrate the method on CdS thin films and benchmark the results against Minkov’s reflectance-based approach and representative literature data.

## 2 Method

When a light beam incident normally passes through a structure consisting of air-thin film–substrate–air, the reflectance equation is given as,^14^

1 $$R\left(k_{0}\right)=\frac{A^{\prime }-\left(B_{1}^{\prime }\cos2\delta -B_{2}^{\prime }\sin2\delta \right)x+C^{\prime }x^{2}}{A^{\prime \prime }-\left(B_{1}^{\prime \prime }\cos2\delta -B_{2}^{\prime \prime }\sin2\delta \right)x+C^{\prime \prime }x^{2}}+\frac{A^{\prime \prime \prime }x^{2}}{A^{\prime \prime }-\left(B_{1}^{\prime \prime }\cos2\delta -B_{2}^{\prime \prime }\sin2\delta \right)x+C^{\prime \prime }x^{2}}\times \frac{1}{C^{\prime \prime }-\left(E_{1}^{\prime \prime }\cos2\delta -E_{2}^{\prime \prime }\sin2\delta \right)x+F^{\prime \prime }x^{2}}$$

where,

2a $$A^{\prime }=\left[{\left(n-1\right)}^{2}+\kappa^{2}\right]\left[{\left(n+n_{s}\right)}^{2}+\kappa^{2}\right]$$

2b $$B_{1}^{\prime }=2\left[\left(n^{2}+\kappa^{2}-1\right)\left(n^{2}+\kappa^{2}-n_{s}^{2}\right)+4\kappa^{2}n_{s}\right]$$

2c $$B_{2}^{\prime }=4\kappa \left[n_{s}\left(n^{2}+\kappa^{2}-1\right)-\left(n^{2}+\kappa^{2}-n_{s}^{2}\right)\right]$$

2d $$C^{\prime }=\left[{\left(n+1\right)}^{2}+\kappa^{2}\right]\left[{\left(n-n_{s}\right)}^{2}+\kappa^{2}\right]$$

2e $$A^{\prime \prime }=\left[{\left(n+1\right)}^{2}+\kappa^{2}\right]\left[{\left(n+n_{s}\right)}^{2}+\kappa^{2}\right]$$

2f $$B_{1}^{\prime \prime }=2\left[\left(n^{2}+\kappa^{2}-1\right)\left(n^{2}+\kappa^{2}-n_{s}^{2}\right)-4\kappa^{2}n_{s}\right]$$

2g $$B_{2}^{\prime \prime }=4\kappa \left[n_{s}\left(n^{2}+\kappa^{2}-1\right)+\left(n^{2}+\kappa^{2}-n_{s}^{2}\right)\right]$$

2h $$C^{\prime \prime }=\left[{\left(n-1\right)}^{2}+\kappa^{2}\right]\left[{\left(n-n_{s}\right)}^{2}+\kappa^{2}\right]$$

2i $$A^{\prime \prime \prime }=64n_{s}{\left(n_{s}-1\right)}^{2}{\left(n^{2}+\kappa^{2}\right)}^{2}$$

2j $$C^{\prime \prime }=\left[{\left(n+1\right)}^{2}+\kappa^{2}\right]\left[\left(n+1\right)\left(n+n_{s}^{2}\right)+\kappa^{2}\right]$$

2k $$E_{1}^{’’}=2\left[\left(n^{2}+\kappa^{2}-1\right)\left(n^{2}+\kappa^{2}-n_{s}^{2}\right)-2\kappa^{2}\left(n_{s}^{2}+1\right)\right]$$

2l $$E_{2}^{’’}=2\kappa \left[\left(n^{2}+\kappa^{2}-n_{s}^{2}\right)+\left(n_{s}^{2}+1\right)\left(n^{2}+\kappa^{2}-1\right)\right]$$

2m $$F^{\prime \prime }=\left[{\left(n-1\right)}^{2}+\kappa^{2}\right]\left[\left(n-1\right)\left(n-n_{s}^{2}\right)+\kappa^{2}\right]$$

and

2n $$x=\exp\left(-\alpha d\right);\alpha =4\pi \kappa k_{0};\delta =2\pi ndk_{0}$$

where *n_s_* is the refractive index of the substrate, *k*_0_ is the wavenumber and *d* is the thickness of the film. Since refractive-index dispersion influences the repetition frequency of the optical interference pattern, *R*(*k*_0_) becomes nonstationary; therefore, the Continuous Wavelet Transform (*CWT*) is an appropriate tool for its analysis. The *CWT* of the reflectance signal can be expressed as

3 $$CWT\left(a,b\right)=\frac{1}{a^{1/2}}\int_{-\infty }^{\infty }\psi^{*}\left(\frac{k_{0}-b}{a}\right)R\left(k_{0}\right)dk_{0}$$

where, $\psi \left(\frac{k_{0}-b}{a}\right)$ is the analyzing wavelet derived from the admissible and localized mother wavelet $\psi \left(k_{0}\right)$ .^21,22^ Here, *b* denotes the translation parameter corresponding to the wavelet’s location, and *a* is the dilation parameter (*a* ≥ 0) related to scale length.^23^ By applying the convolution theorem,^24^ eq 16 can be rewritten in the Fourier domain (*x*_0_) as

4 $$CWT\left(a,b\right)=a^{1/2}\int_{-\infty }^{\infty }{\mathrm{\psi ̂}}^{*}\left(ax_{0}\right)\mathrm{R̂}\left(x_{0}\right)\exp\left(ibx_{0}\right)dx_{0}=IFT\left\{{\mathrm{\psi ̂}}^{*}\left(ax_{0}\right)\mathrm{R̂}\left(x_{0}\right)\right\}$$

where $a^{1/2}\mathrm{\psi ̂}\left(ax_{0}\right)$ and ***R*** (*x*_0_) represent the Fourier Transform (FT) of $a^{-1/2}\psi \left(\frac{k_{0}}{a}\right)$ and *R*(*k*_0_), respectively. This representation allows the wavelet transform to be efficiently evaluated in the Fourier domain by means of the inverse Fourier transform (*IFT*) algorithm.

The Paul wavelet was selected as the analyzing wavelet owing to its tunable joint resolution in the real (*k*_0_) and Fourier (*x*_0_) domains. The corresponding expressions in the *k*_0_ and *x*_0_ spaces are presented in eqs 17 and 18, respectively,

5 $$\psi_{a,b}\left(k_{0}\right)=\frac{1}{\sqrt{a}}\frac{2^{m}m!{\left(1-i\left(\frac{k_{0}-b}{a}\right)\right)}^{-\left(m+1\right)}}{2\pi \sqrt{\left(2m\right)!/2}}$$

6 $$\mathrm{\psi ̂}\left(ax_{0}\right)=\frac{2^{m}}{\sqrt{m\left(2m-1\right)!}}\left(ax_{0}{)}^{m}\exp(-ax_{0}\right)\xi \left(x_{0}\right)$$

where *m* denotes the order of the Paul wavelet and *ξ*(*x*_0_) is the Heaviside function.^22,25^ The Paul wavelet belongs to the class of progressive wavelets, making it well suited for analyzing causal signals because $\mathrm{\psi ̂}\left(ax_{0}\right)=0$ for *x*_0_ < 0.^26^ The center and variance of the analyzing wavelet in *k*_0_ domain are $k_{0_{C}}=b$ and ${\left(\Delta k_{0}\right)}^{2}=\frac{a^{2}}{2m-1}$ , whereas in *x*_0_ domain they are $x_{0_{C}}=\frac{2m+1}{2a}$ and ${\left(\Delta x_{0}\right)}^{2}=\frac{2m+1}{4a^{2}}$ , respectively. The degree *m* determines the trade-off between the resolutions of the two domains. By exploiting the localization property,^27^ and assuming that *n*(*k*_0_) remains approximately constant, *n*(*k*_0_) ≅ *n*(*b*) within the interval $\left[k_{0_{C}}-\Delta k_{0},k_{0_{C}}+\Delta k_{0}\right]$ , the Fourier transform of *R*(*k*_0_) is obtained as,

7 $$\mathrm{R̂}\left(x_{0}\right)=C_{1}\delta \left(x_{0}+4\pi D\right)+C_{2}\delta \left(x_{0}\right)+C_{3}\delta \left(x_{0}-4\pi D\right)$$

*C*_1_, *C*_2_, and *C*_3_ are constants. Using $\mathrm{\psi ̂}\left(ax_{0}\right)=0$ when *x*_0_ ≤ 0, eq 17 is reduced by inserting eqs 19 and 20,

8 $$CWT\left(a,b\right)=\sqrt{a}\int_{-\infty }^{\infty }C_{1}\delta \left(x_{0}-4\pi D\right)\frac{2^{m}}{\sqrt{m\left(2m-1\right)!}}\left(ax_{0}{)}^{m}\exp(-ax_{0}\right)\exp\left(ibx_{0}\right)dx_{0}$$

and the squared normalized modulus of *CWT*(*a*,*b*) was calculated as,

9 $${|CWT\left(a,b\right)|}^{2}=a{\mathrm{\psi ̂}}^{2}\left(aX\right){\alpha_{1}}^{2}=\frac{2^{2m}}{m\left(2m-1\right)!}a\left(a4\pi D{)}^{2m}\exp(-a8\pi D\right)$$

This has a matrix of *a* × *b* dimensions. eq 22 peaks at *a_max_* for a fixed *b* according to eq 22,

10 $$a_{max}=\frac{2m+1}{8\pi D},\to n=\frac{2m+1}{8\pi da_{max}}$$

Determination of the spectral dispersion of *n*(*k*_0_) requires prior knowledge of the film thickness. Once the dispersion of *n*(*k*_0_) is obtained, the extinction coefficient *κ*(*k*_0_) can be determined from the envelopes of the maxima *R_M_* or minima *R_M_* of the reflection spectrum using eq 24 ^14^

11 $$R_{M},R_{m}=\frac{A^{\prime }\pm B_{1}^{\prime }x+C^{\prime }x^{2}}{A^{\prime \prime }\pm B_{1}^{\prime \prime }x+C^{\prime \prime }x^{2}}+\frac{A^{\prime \prime \prime }x^{2}}{\left(A^{\prime \prime }\pm B_{1}^{\prime \prime }x+C^{\prime \prime }x^{2}\right)\left(C^{\prime \prime }\pm E_{1}^{\prime \prime }x+F^{\prime \prime }x^{2}\right)}$$

where + in the ± refers to *R_M_* and – to *R_M_*.

The Paul-wavelet method relies on the presence of well-defined interference fringes in the reflectance spectrum; therefore, when strong absorption suppresses these fringes, the method does not provide sufficient information for reliable retrieval of optical parameters. In such cases, alternative model-based approaches, such as Cauchy or Sellmeier dispersion relations, may be used to estimate the optical constants through fitting procedures.

### 2.1 Simulation Studies

To demonstrate the applicability of the proposed method, a simulation study was conducted. The reflectance spectrum *R*(*k*_0_) was calculated over the wavenumber range [0.91–2.22] μm^‑1^ using eq 1 with the parameters defined in eq 25a–28d. The computing *R*(*k*_0_) curve is shown in Figure 1a,

12a $$n\left(k_{0}\right)=A+B{k_{0}}^{2}+C{k_{0}}^{4}$$

12b $$A=2.60,B=3.00\times 10^{-1}\mu m^{2},C=2.00\times 10^{-4}\mu m^{4}$$

12c $$\kappa \left(k_{0}\right)=\left(\frac{10^{3}}{4\pi k_{0}}\right)10^{\left(1.5\times {k_{0}}^{2}\right)-8}$$

12d $$n_{0}=1,n_{s}=1.51,d=3\mu m$$

**Figure 1:**
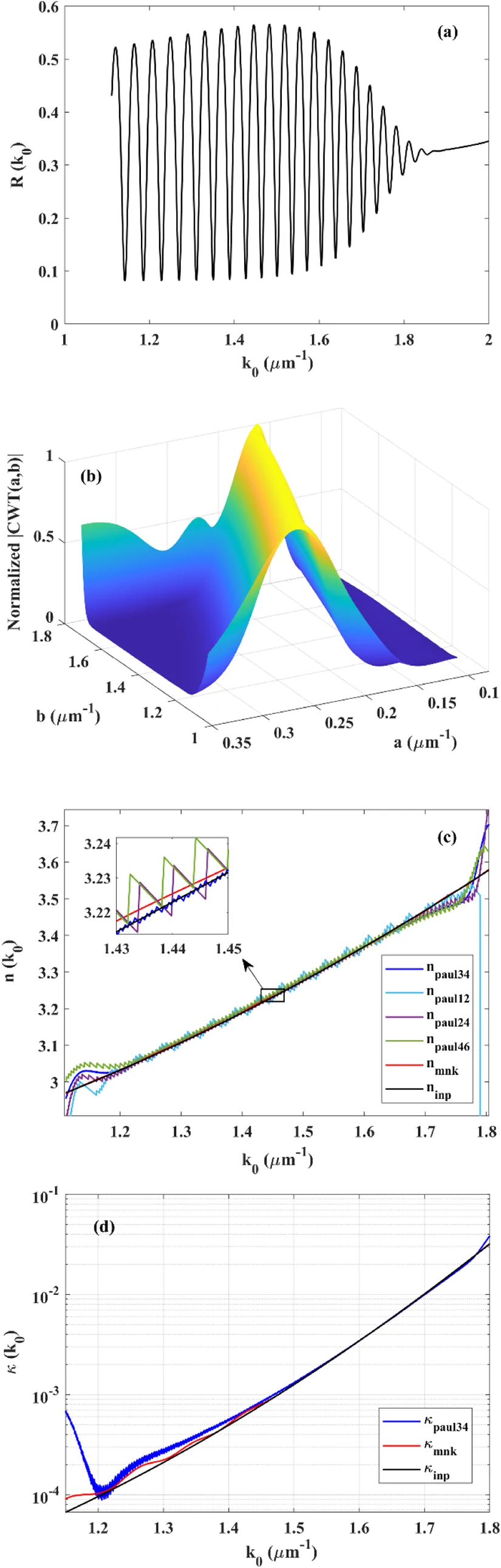
(a) Theoretically generated *R*(*k*_0_). (b) The normalized modulus obtained from the Paul wavelet analysis (*m* = 34). (c) *n*(*k*_0_) curves determined by the Paul wavelet analysis with different *m* values (*m* = 12–cyan, m = 24–purple, *m* = 34–blue, and *m* = 46–green curves), compared with the Minkov method (red) and the presumed reference curve (black). The inset illustrates the accuracy of the calculated results. (d) *κ*(*k*_0_) curves determined by the Paul wavelet analysis (*m* = 34–blue), the Minkov method (red), and the presumed reference curve (black).

The simulated reflectance signal was analyzed using the Paul wavelet with *m* = 34, and the resulting normalized modulus is presented in Figure 1b. The spectral dispersion of *n*(*k*_0_), calculated from eq 23, is shown in Figure 1c. The refractive index *n*(*k*_0_) was obtained as a continuous function, and the corresponding three-term Cauchy parameters derived from the fitting process are listed in Table 1. The accuracy of the results was assessed through a mean relative difference analysis,

13 $$\overset{®}{\delta n/n}=\left|n_{cal}-n_{inp}\right|/n_{inp}$$

yielding an average relative error of 7.64 × 10^‑4^. The sharp deviations observed at the edges of the calculated *n*(*k*_0_) curve originate from edge effects intrinsic to the method, which arise due to the finite-length nature of the *R*(*k*_0_) signal.^28^ In wavelet-based analysis, methods such as padding^29^ or the Meyer approach^23^ are commonly proposed in the literature to mitigate edge effects. However, since these methods introduce artificial extensions or modifications to the measured signal, they were not employed in this study in order to preserve the physical integrity of the data.

**Table 1:** Three-Term Cauchy Parameters (Computed and Input) and Corresponding Mean Relative Difference Results for Refractive Index Dispersions

			A	B (μm^2^)	C (μm^4^)	$\overset{®}{\delta n/n}$
Simulation Study	Inputs		2.60	3.00 × 10^‑1^	2.00 × 10^–4^	0
	Paul (*m*)	12	2.58	3.30 × 10^‑1^	–8.51 × 10^–3^	2.76 × 10^–3^
		24	2.57	3.24 × 10^‑1^	–8.74 × 10^–3^	2.37 × 10^–3^
		34	2.59	3.07 × 10^‑1^	–1.31 × 10^–3^	2.15 × 10^–4^
		46	2.58	3.24 × 10^‑1^	–5.37 × 10^–3^	2.31 × 10^–3^
	Minkov		2.61	2.92 × 10^‑1^	6.92 × 10^–4^	3.73 × 10^–4^
Noisy Signal Study	Paul (*m*)	34	2.55	3.35 × 10^‑1^	–1.38 × 10^–2^	1.73 × 10^–3^
	Minkov		3.56	–2.55 × 10^‑1^	9.53 × 10^–2^	5.58 × 10^–2^
Experimental Study	Paul (*m*)	32	1.72	5.82 × 10^‑2^	–4.38 × 10^–2^	-
	Minkov		2.60	2.97 × 10^‑1^	6.92 × 10^–4^	-

Instead, the reliability of the results is ensured by focusing on the central spectral region, where the wavelet coefficients are fully supported within the signal window. This region provides the most reliable estimates of the optical constants, while boundary regions are excluded due to reduced wavelet support. This treatment is consistent with the cone of influence (COI) concept in wavelet analysis, defined here using the e-folding time of Torrence and Compo,^20^

14 $$\tau \left(a\right)=\frac{a}{\sqrt{2}}$$

which, together with the ridge scale *a_max_* from eq 23, gives the reliable-region boundaries,

15 $$b_{left}=k_{0,min}+\tau \left(a_{max}^{left}\right),b_{right}=k_{0,max}-\tau \left(a_{max}^{right}\right)$$

For the simulation study (*m* = 34, *d* = 3 μm), this yields *b_left_* = 1.33 μm^–1^ and *b_right_* =1.62 μm^–1^, consistent with the edge deviations in Figure 1c, d. To further investigate the influence of resolution control, the *R*(*k*_0_) signal was additionally analyzed using the Paul wavelet with various *m* values (m = 12, 24, 34, 46 For comparison, the same signal was also analyzed using the empirical method proposed by Minkov.^14^ All corresponding three-term Cauchy coefficients and mean relative differences obtained from both approaches are summarized in Table 1.

Once *n*(*k*_0_) has been determined, the extinction coefficient *κ*(*k*_0_) can be subsequently obtained. In this work, *κ*(*k*_0_) was computed from the analytical expression derived in the proposed framework, whereas the Minkov method retrieves both *n*(*k*_0_) and *κ*(*k*_0_) simultaneously via the Newton–Raphson iterative scheme. A comparison between the *κ*(*k*_0_) curves obtained from the Paul wavelet approach (*m* = 34), the Minkov method, and the input data is presented in Figure 1d. The simulation results illustrate the influence of the *m* parameter in the Paul wavelet analysis and identify *m* = 34 as the optimal resolution parameter for the theoretically generated *R*(*k*_0_) spectrum. The mean relative difference values and the three-term Cauchy parameters show that the *n*(*k*_0_) obtained by the Paul wavelet and Minkov methods are in very good agreement. The Paul wavelet analysis with *m* = 34 provides slightly higher accuracy compared with the Minkov method, yielding results that closely match the presumed reference curve. In contrast, for the *κ*(*k*_0_), the Minkov method yields slightly better accuracy than the Paul-wavelet approach, which can be attributed to the idealized nature of the simulated reflectance data.

The simulation study was extended to include a noisy-signal analysis in order to evaluate the performance of the enhanced Paul-wavelet method. Figure 2a shows the noisy *R*(*k*_0_) spectrum obtained after adding 10% random noise to the signal presented in Figure 1a. The corresponding normalized modulus and the determined *n*(*k*_0_) and *κ*(*k*_0_) dispersions (*m* = 34) are shown in Figure 2b–d, respectively. In addition, the noisy signal was also analyzed using the Minkov method, and the resulting three-term Cauchy parameters along with the mean relative difference values are summarized in Table 1. Figure 2c and Table 1 show that, in the analysis of the noisy signal, the *n*(*k*_0_) values determined by the Minkov method exhibit significant deviations, whereas those obtained from the Paul-wavelet method (*m* = 34) demonstrate good agreement with the presumed reference curve. By contrast, the Minkov method relies on the extrema of the signal envelope, leading to the observed deviations under noisy conditions. Consequently, the analysis confirms that the Paul-wavelet method possesses strong *noise immunity* owing to its repetition-frequency-based analysis. Applying the same COI criterion (eqs 30–31) to the noisy-signal analysis yields boundaries nearly identical to the noise-free case (*b_left_* = 1.33 μm^‑1^, *b_right_* = 1.62 μm^‑1^), confirming that the extent of the reliable spectral region is governed by the ridge scale *a*_*max*_rather than by the noise level. Figure 2d further indicates that the retrieval of *κ*(*k*_0_) is inherently more sensitive to noise than that of *n*(*k*_0_), since *κ* is governed primarily by the *absolute reflectance level* (i.e., fringe amplitude), which is directly perturbed by additive random noise. Accordingly, the Minkov-derived *κ*(*k*_0_) exhibits pronounced oscillations, particularly in the low *k*_0_ region, reflecting the instability of envelope-extrema extraction under noisy conditions. In contrast, the Paul-wavelet-based *κ*(*k*_0_) remains comparatively smooth and preserves the overall trend of the presumed reference curve.

**Figure 2:**
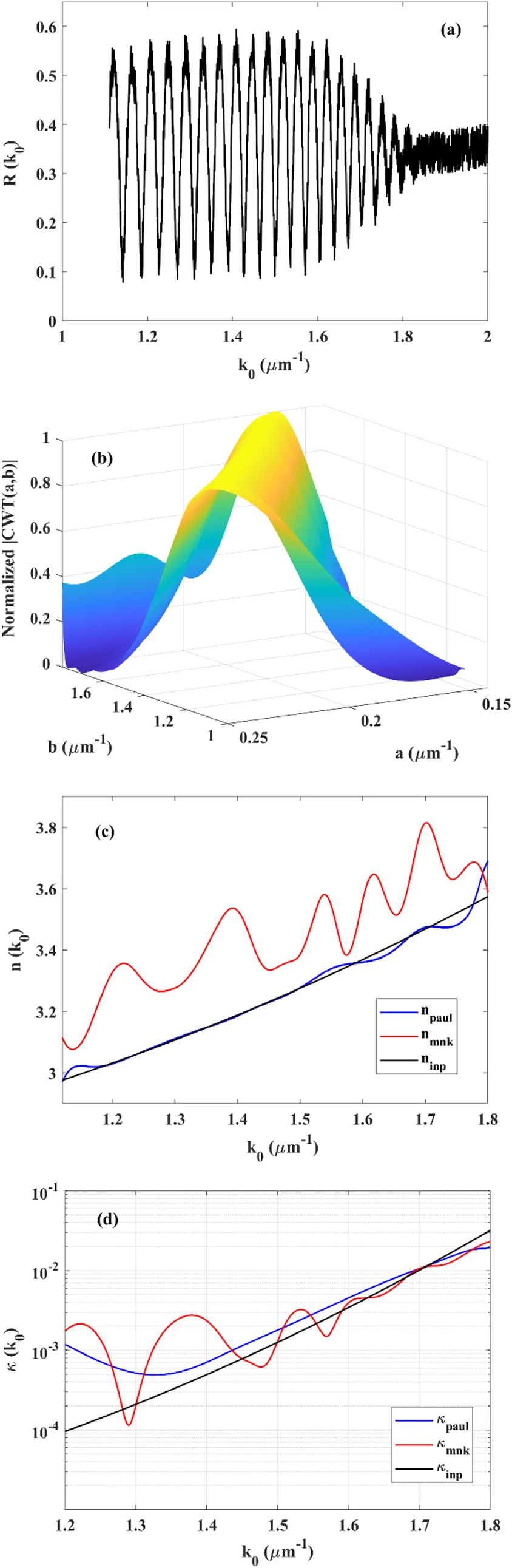
(a) The theoretically generated noisy *R*(*k*_0_) (10% noise added). (b) The normalized modulus of the Paul wavelet analysis (*m* = 34). (c) *n*(*k*_0_) curves determined by the Paul wavelet analysis (*m* = 34–blue), the Minkov method (red), and the presumed reference curve (black). (d) *κ*(*k*_0_) curves determined by the Paul wavelet analysis (*m* = 34–blue), the Minkov method (red), and the presumed reference curve (black).

## 3 Experimental Studies

The Cd–S thin films were deposited on soda-lime glass (SLG) by using an RF and DC sputtering system in which CdS was used as target. The total sputtering time was determined as 25 min to obtain the desired thickness of thin films. The individual deposition layer thicknesses were controlled by a quartz crystal connected to the Inficon XTM/2 Deposition monitor. The substrate temperature was kept at room temperature. Then, CdS films were annealed under a nitrogen atmosphere at 100 °C for 30 min to improve the crystallinity of the deposited films. A JSM-600 scanning electron microscope (SEM) equipped with an energy dispersive X-ray spectroscopy (EDS) detector facility operated at 20 kV was used to determine the chemical composition of the deposited films. The EDS analysis result shows that the stoichiometry of the films is nearly a 1:1 ratio as desired (see Figure 3a). EDS analysis was carried out with SEM system and the observed spectrum is shown in Figure 3a. The relevant atomic ratios of the elements are 51% S and 49% Cd after annealing treatment. The results reveal that the CdS film is nearly stoichiometric within the experimental error of ±2%. A SEM image for a typical sample is shown in Figure 3b, and it is seen from the figure that the film has crack-free and grainy surface morphology. It shows that the distribution of grains over the surface of the films is quite uniform and regular. X-ray diffraction (XRD) measurements were performed by the Rigaku Miniflex XRD system equipped with a Cu Kα radiation source having wavelength of 1.54 Å, and the result is presented in Figure 3c. The obtained X-ray diffraction pattern for the film showed that the main orientation of the CdS thin film was found to be along the (111) direction at 2θ ∼ 26.5°, and the deposited films have a cubic crystal structure and lattice parameters of this structure are *a* = *b* = *c* = 5.689 Å according to the card no of JCPDS (joint committee on powder diffraction standards) 78–1922. The average microcrystalline grain size *GS* was estimated from the XRD pattern using the Scherrer’s formula,

16 $$GS=\frac{K\lambda_{0}}{\beta \cos\theta }$$

where *λ*_0_ is the wavelength of the X-ray radiation source, *β* is the full width of half-maximum value of a diffraction peak, *θ* is the diffraction angle, and *K* is the Scherrer’s constant in the order of unity for usual crystal structure; and in this study, due to cubic symmetry, *K* was taken as 0.94.^30^ The calculated grain size value is about 34 nm.

**Figure 3:**
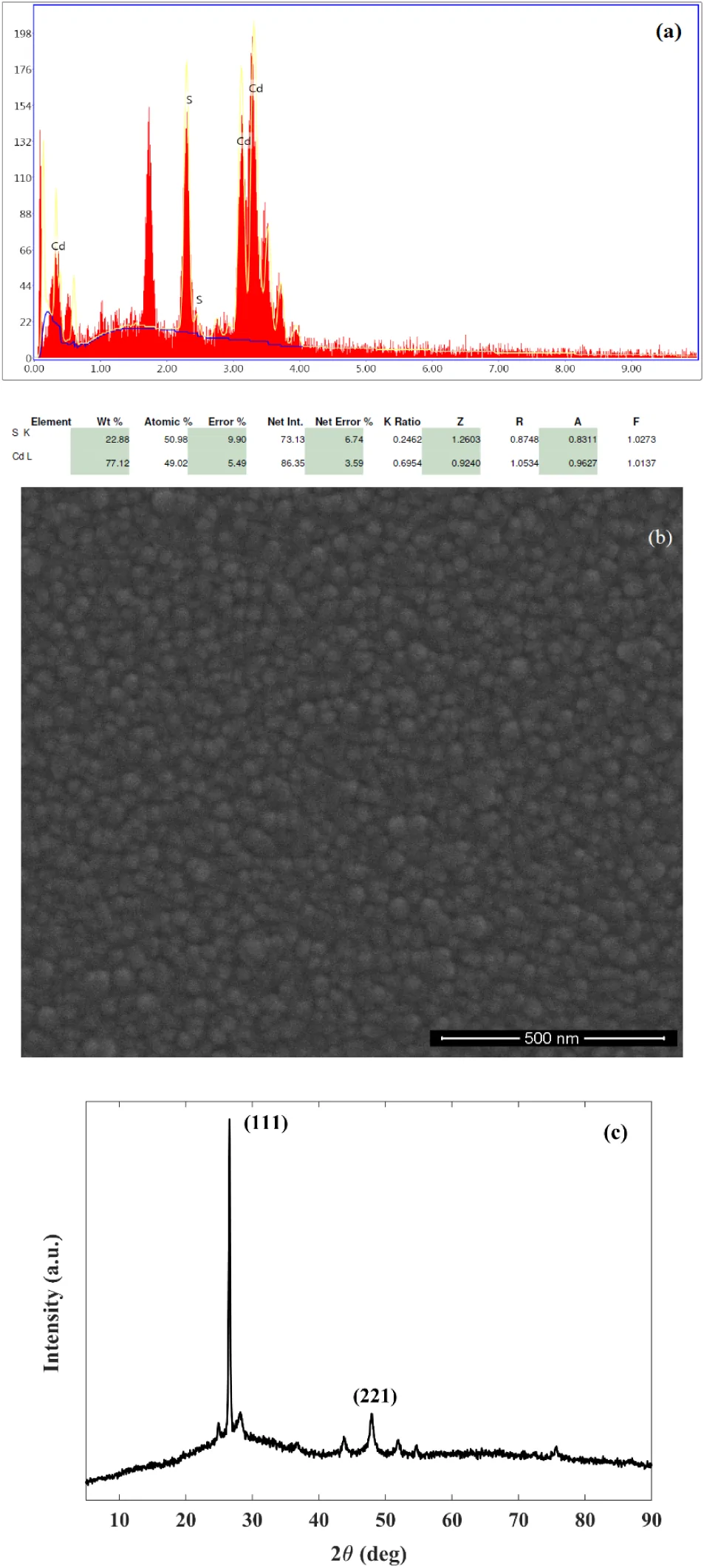
(a) EDS analysis results of the CdS film. (b) SEM micrograph of the CdS film. (c) X-ray diffraction pattern of the CdS film.

## 4 Results and Discussion

### 4.1 Paul Wavelet Analysis of the Experimental Data

A CdS thin film was used as a test specimen to validate the proposal method. Reflectance measurements were performed using an MS260i spectrograph equipped with an integrating sphere. The light source was a 10–250 W quartz–tungsten–halogen lamp, and the grating had 1800 lines/mm. Reflected light was detected by a charge-coupled device (CCD), and the entire measurement was computer-controlled. The film thickness was measured using a Dektak profilometer and found to be *d* = 461 ± 10 nm. To quantify the effect of film thickness uncertainty on the retrieved optical constants, a sensitivity analysis was performed by varying the thickness within its experimental uncertainty range (*d* = 461 ± 10 nm). The corresponding variations in the refractive index were used to define a wavelength-dependent sensitivity function as $S_{n}\left(k_{0}\right)=\frac{n_{max}\left(k_{0}\right)-n_{min}\left(k_{0}\right)}{2n_{ref}\left(k_{0}\right)}$ , where *n*_max_(*k*_0_) and *n*_min_(*k*_0_) denote the refractive index values obtained at the upper and lower bounds of the thickness variation, respectively. The mean sensitivity over the investigated spectral range was found to be ${\mathrm{S̅}}_{n}=0.0216$ . Similarly, the extinction coefficient sensitivity was defined as $S_{\kappa }\left(k_{0}\right)=\frac{\kappa_{max}\left(k_{0}\right)-\kappa_{min}\left(k_{0}\right)}{2\kappa_{ref}\left(k_{0}\right)}$ , and its mean value was calculated as ${\mathrm{S̅}}_{\kappa }=0.3105$ (see Figure 4c and d). At room temperature, the reflectance spectrum of the specimen was recorded in the wavenumber range [0.97 – 2.00] μm^‑1^, with a sampling interval of Δ*k*_0_ ≈ 2 × 10^‑4^ μm^‑1^ (Figure 4a). The measured *R*(*k*_0_) signal was analyzed using the Paul wavelet method (*m* = 32), where the wavelet decomposition parameter *m* was selected via an automated scan over predefined candidate values (*m* = 8–42). For each candidate *m*, the refractive-index dispersion *n*(*k*_0_) was reconstructed and its discrete total variation (TV), which measures the cumulative variation between adjacent data points, was calculated as^31^

17 $$TV=\overset{N-1}{\underset{i=1}{\sum }}\left|n\left(k_{0,i+1}\right)-n\left(k_{0,i}\right)\right|$$

**Figure 4:**
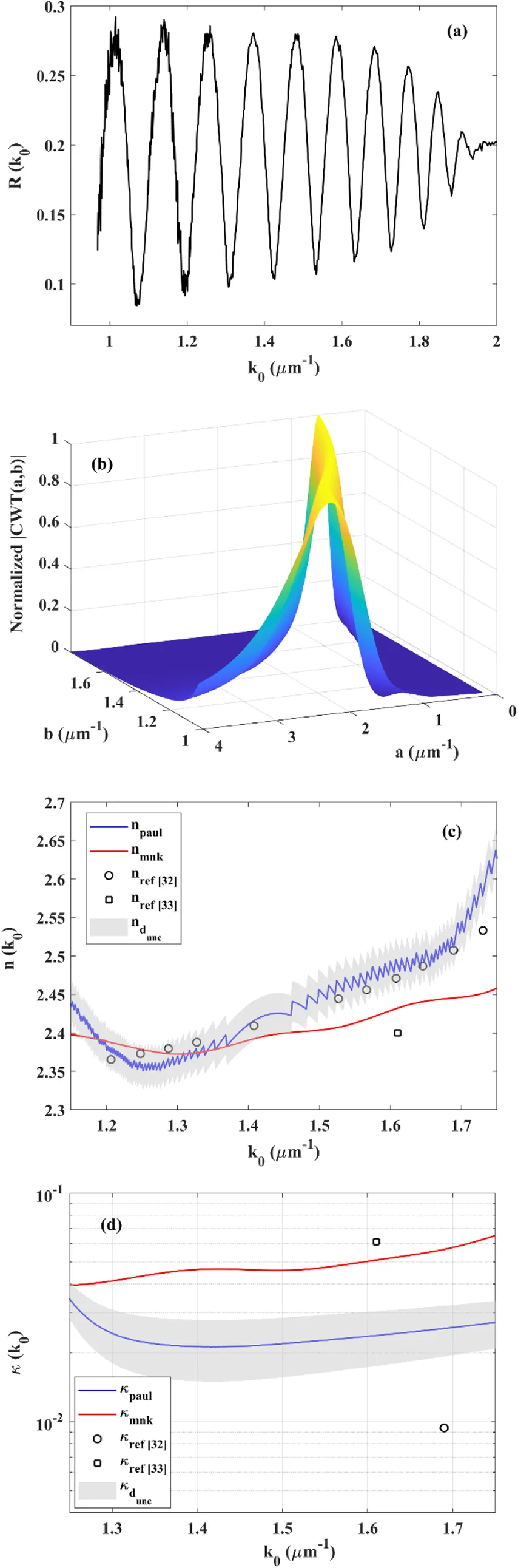
(a) Measured *R*(*k*_0_), of the CdS thin film. (b) The normalized modulus of the Paul wavelet analysis (*m* = 32). (c) *n*(*k*_0_) curves determined by the Paul wavelet analysis (*m* = 32–blue), the Minkov method (red); circles and squares indicate the literature values reported in refs 32,33 (black), respectively. The shaded region represents the uncertainty band in the retrieved *n*(*k*_0_) induced by the experimental film thickness variation (*d* = 461 ± 10 nm). (d) *κ*(*k*_0_) curves determined by the Paul wavelet analysis (*m* = 32–blue), the Minkov method (red); circles and squares indicate the literature values reported in refs 32,33 (black), respectively. The shaded region represents the uncertainty band in the retrieved *κ*(*k*_0_) induced by the experimental film thickness variation (*d* = 461 ± 10 nm).

The optimal wavelet order was defined as the value yielding the minimum TV, thereby suppressing artificial oscillations in the retrieved refractive index dispersion.

The corresponding normalized modulus, as well as the *n*(*k*_0_) and *κ*(*k*_0_) dispersions, were obtained and are presented in Figure 4b–d, respectively. The computed three-term Cauchy parameters are listed in Table 1. For comparison, the same reflectance signal was also analyzed using the Minkov method, and the resulting *n*(*k*_0_) and *κ*(*k*_0_) curves are included in Figure 4c and d, respectively. The corresponding Cauchy parameters derived from the Minkov method are also summarized in Table 1. The experimental study results prove that the *n*(*k*_0_) dispersion determined by the Paul wavelet method has high harmony with the *n*(*k*_0_) values presented in ref 32,33 . On the other hand, the *n*(*k*_0_) dispersion determined by the Minkov method has a small shift on the curve. This situation may be due to the dispersion coefficient (the *B* parameter in the Cauchy equation) being calculated with a small error caused by noise in the reflectance spectrum. The Paul-wavelet analysis yields *κ*(*k*_0_) values that differ markedly from those reported in refs 32,33 . This discrepancy is plausible because *κ* is primarily governed by absorption and is therefore highly sensitive to deposition-dependent variations in microstructure and band-edge characteristics.^34^ Moreover, *κ* extraction from reflectance is generally more sensitive to measurement noise than *n*, which can further amplify differences with literature data. For the CdS film, the smaller thickness (*d* = 461 nm) gives a larger *a_max_* near *k*_0,*min*_ (via eq 23), making the COI criterion (eqs 30–31) overly conservative here; *b_left_* falls beyond the measured window (*k*_0,*max*_ = 2.00 μm^‑1^). We report this for completeness; the edge deviations in Figure 4c, d are nonetheless interpreted qualitatively, as in Figures 1 and 2, consistent with the good agreement of *n*(*k*_0_) with literature values (refs 32,33 ).

### 4.2 Error Analysis

The refractive index error analysis of the enhanced Paul wavelet approach was examined. The relative error on the refractive index (Δnn) depending on eq 23 is given as

18 $$\frac{\Delta n}{n}=\left[{\left({\left(\frac{\Delta a_{\max}}{a_{\max}}\right)}^{2}+{\left(\frac{\Delta d}{d}\right)}^{2}\right)}^{1/2}\right]$$

The dilation (scale) parameter is discretized as,^20^

19 $$a_{j}=\frac{\pi }{x_{02}}2^{jdj},dj=\frac{\log_{2}\left(x_{02}/x_{01}\right)}{N},j=0,1,...,N$$

where *x*_01_ and *x*_02_ are the lower and upper bounds of the Fourier transform of the reflectance spectrum (i.e., where $\mathrm{R̂}\left(x_{0}\right)\to 0$ );^25^ and *N* is the total number of the scale.

The relative error of the *a_max_* is computed as,

20 $$\frac{\Delta a_{max}}{a_{max}}={\left(\frac{x_{02}}{x_{01}}\right)}^{1/N}-1.$$

For this work, *N* = 1000, *x*_01_ = 0.95 μm and *x*_02_ = 1.99 μm values were obtained from $\mathrm{R̂}\left(x_{0}\right)$ and the relative error of the scale was computed as,

21 $$\frac{\Delta a_{max}}{a_{max}}=0.0066.$$

The absolute error of the thickness is equal Δ*d* = 1.00 × 10^‑2^ μm, and the relative error of the thickness was calculated as $\frac{\Delta d}{d}=0.024$ . The relative error of the improved Paul wavelet method is $\frac{\Delta n}{n}=0.025$ , which is consistent with the literature.^27^

## 5 Conclusion

Using the reflectance spectrum, the Paul wavelet approach was enhanced to calculate the *n*(*k*_0_) dispersion and, subsequently, the *κ*(*k*_0_) dispersion within the same framework. Simulation studies verified that the retrieved *n*(*k*_0_) is continuous and highly accurate, as supported by the three-term Cauchy parameters and the mean relative difference results in Table 1. The method provides explicit control over resolution, and the simulations highlight the practical importance of this tunability. The noise immunity arising from repetition-frequency-based analysis was confirmed in the noisy signal study $\left(\overset{®}{\delta n_{noise}/n_{noise}}=7.71\times 10^{-4}\right)$ , where envelope-extrema-based analysis becomes more vulnerable to noise.

Experimental validation on a CdS thin film further demonstrated that the *n*(*k*_0_) dispersion obtained by the proposed approach is consistent with literature results, while *κ*(*k*_0_) shows larger variability, which is plausible given its strong dependence on absorption-related, deposition-sensitive band-edge characteristics and its higher sensitivity to measurement noise in reflectance-based extraction. One limitation of the technique is the requirement to ascertain film thickness, which is shared by all interference-based signal analysis methods.

The method is most suitable for moderately to strongly absorbing films (typically in the range of approximately (10^2^ cm^‑1^ < *α* < 10^5^ cm^‑1^), including opaque, colloidal, or heavily doped semiconductor layers where transmission-based analysis is not feasible. For very weakly absorbing films (*α* < 10^2^ cm^‑1^), ellipsometric or photothermal techniques may be more appropriate, as κ retrieval becomes increasingly sensitive to noise in the reflectance signal in this regime.
